# Primary cutaneous actinomycosis: a diagnosis consideration in people living with HIV/AIDS

**DOI:** 10.1186/s12981-019-0232-4

**Published:** 2019-07-30

**Authors:** Priyatam Khadka, Soniya Koirala

**Affiliations:** 1 Department of Microbiology, Sumeru Hospital, Lalitpur, Nepal; 2 Tribhuvan University, Tri-Chandra Multiple Campus, Ghantaghar, Kathmandu, Nepal; 3 Department of Dermatology and Venerology, Tribhuvan University Teaching Hospital, Kathmandu, Nepal

**Keywords:** Diagnostic consideration, Penicillin, Primary cutaneous actinomycosis, AIDS

## Abstract

**Background:**

Owing to similar clinical presentations, as of cutaneous disease of different etiologies, and extreme rarity in the global incidence; primary cutaneous actinomycosis often remains as diagnostic challenges.

**Case presentation:**

Herein, we describe a case of primary cutaneous actinomycosis, erroneously treated as cutaneous tuberculosis, in a patient living with AIDS. On clinical examination, the characteristic lesion, resembling cutaneous tuberculosis, observed on the dorsum of a left leg. No other lesion elsewhere on the body was observed, however. Cytological examinations of the stabbed biopsy were negative for malignant cells; although hyper-keratosis and mild-acanthosis of epidermis, acute inflammatory infiltrates comprising plasma cell, macrophages and neutrophils were observed in the upper and mid dermis. The pus aspirated from lesion grew a molar tooth, adherent colonies in microaerophilic condition. Further, identifications and susceptibility pattern against recommended antibiotics were assessed as per the CLSI (Clinical and Laboratory Standard Institute) guidelines. Subsequently, the case was then, diagnosed as primary cutaneous actinomycosis. Radiographic imaging of abdomen and lungs were normal; no feature of disseminated actinomycosis seen. Penicillin G followed by Penicillin V, was prescribed for 12 months. The patient underwent progressive changes and no relapse noted on periodic follow-up.

**Conclusion:**

The case underscores cutaneous actinomycosis requires a diagnosis consideration, especially in People Living with HIV/AIDS (PLHA), where myriad of opportunistic cutaneous infections are common.

## Background

Actinomycosis is an unusual sub-acute or chronic suppurative and granulomatous bacterial infection characterized by multiple abscesses, tissue fibrosis, and the formation of sinuses and fistulae [1]. The culprits ensuing the infection, *Actinomyces* spp., are the aerobic or micro-aerophilic filamentous gram-positive bacilli which basically colonized in oropharynx, gastrointestinal tract and uro-genital tract [1–3]. Despite, the indigenous habitat of the pathogen, a few cases of actinomycosis inflicting bone and joints, skin and soft tissue, CNS, respiratory tract, digestive tract can be found on a literature search [1, 3]. Of reported clinical manifestations, disseminated forms, originating from other colonized sites, are likely occurring; however, primary actinomycosis involving only principle site i.e. extremities is extremely rare [3–5].

It is somewhat surprising, the reported incidence of actinomycosis in PLHA has remained low; nevertheless, the persistent impairment in both cellular and humoral immunity are more obvious, due to HIV (human immunodeficiency virus) [6]. The reason for this is not clear, nonetheless, can be speculated due to misdiagnosis. The misdiagnosis often occurs, particularly in PLHA where myriads of other infections are more common, owing to similar indolent and non-specific clinical manifestations of actinomycosis which masquerades as infections of other etiologies [6, 7]. With this backdrop, herein, we report a case of primary cutaneous actinomycosis, mimicking as cutaneous tuberculosis, in a patient living with HIV/AIDS.

## Case presentation

**Fig. 1:**
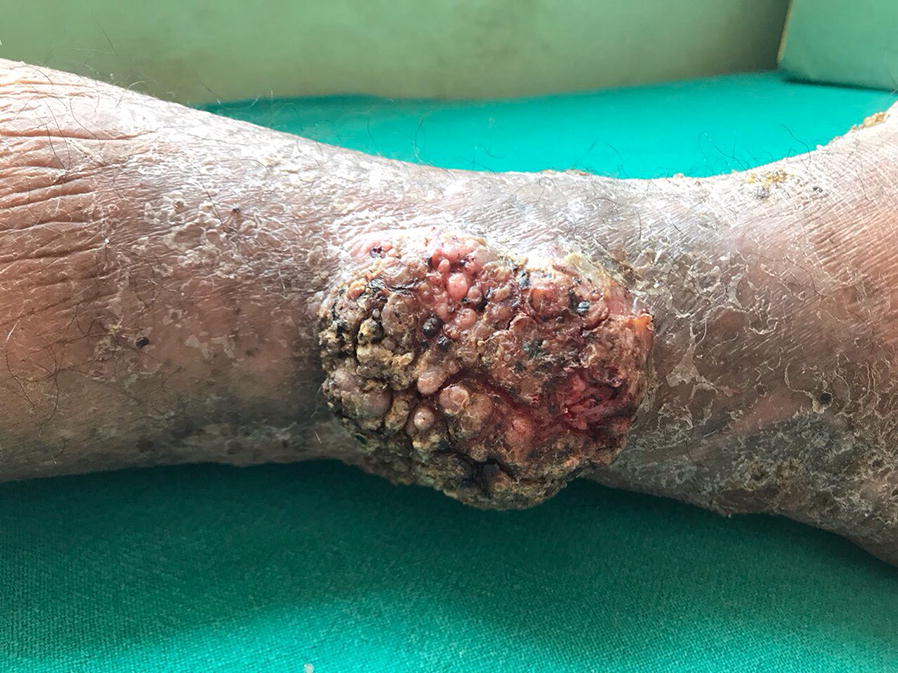
Large plaque-like lesion about 10 cm × 6 cm with papules and nodules; multiple discharging sinuses draining sero-sanguinous fluid

A 42-year-old farmer presented to the Dermatology out-patient department (OPD), Sumeru Hospital, a tertiary care hospital in Kathmandu, with the complaint of a large hard lesion that with time multiplied gradually and had multiple openings discharging pus. He had lived with HIV for 6 years and was from middle-class socio-economic status. Besides, no previous history of systemic illness and major surgical interventions was described. Although, he reported being bitten by own dog earlier 1 month ago on same foot, before the lesion first appears. Based upon the clinical presentations and unresolved lesion with extended courses of antimicrobial therapy (cloxacillin), the previous diagnosis was made as cutaneous tuberculosis, from the local hospital; was treated with anti-tubercular treatment (ATT) for 4 months. Inconsistently, the lesions continued to progress with pus and bloody discharges. On clinical examination, the patient had a large plaque-like lesion about 10 cm × 6 cm overlying skin with papules and nodules on the dorsum of a left leg (Fig. 1). Over the lesion, multiple discharging sinuses draining sero-sanguinous fluid were scattered. No other lesion elsewhere on the body was observed, however. His neurovascular status of the foot was normal with no associated regional lymphadenopathy. Scrutinizing these clinical presentations and clinical history, the differential diagnosis of rifampicin-resistant cutaneous tuberculosis, mycetoma/madura foot, and cutaneous nocardiosis was made.

## Investigation

Histopathological examination, of stabbed biopsy, revealed hyper-keratosis and mild-acanthosis of the epidermis, while acute inflammatory infiltrates comprising plasma cell, macrophages and neutrophils were observed in the upper and mid dermis. Peripheral blood smear portrayed normal cell morphology, normal hemoglobin level (13 gm/dL); while, WBC (12,400/mL) and platelets (4, 15,200/µL) count were slightly elevated. Serological test for HBsAg and HCV were non-reactive; although, HIV was positive (ELISA) with drop off CD4 count 128 cell/µL.

**Fig. 2:**
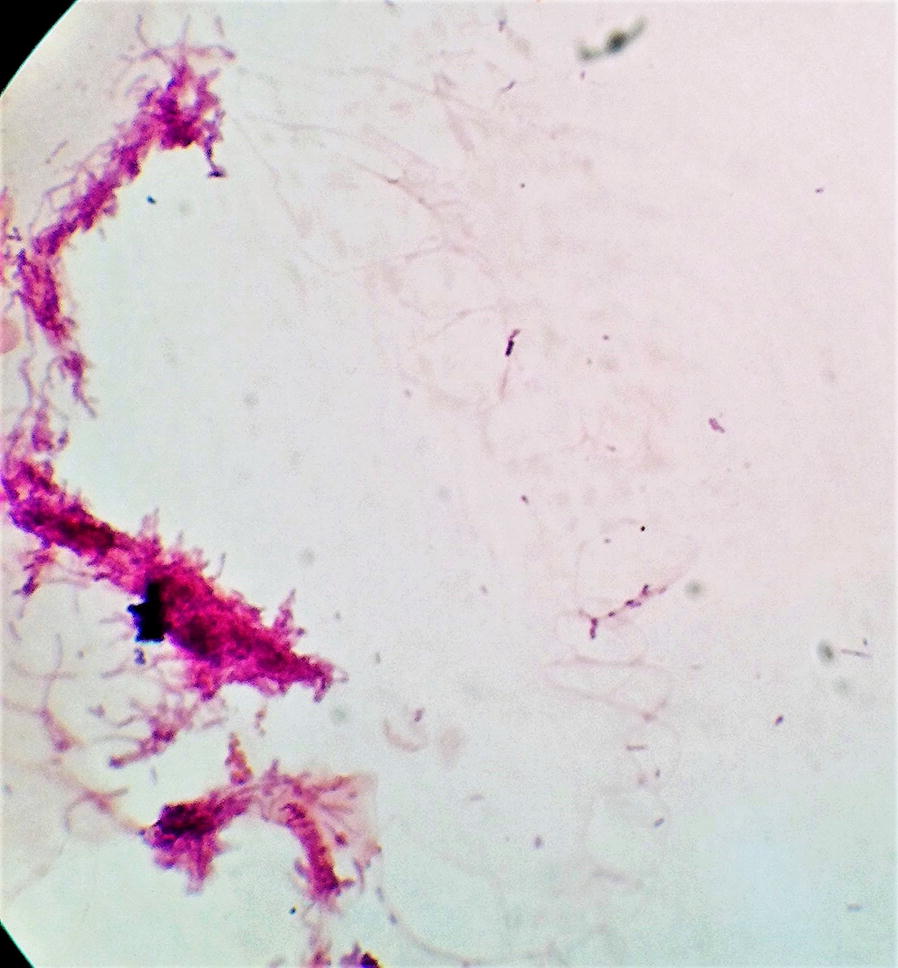
Gram stain: gram positive branching filamentous bacteria suggestive *Actinomyces* spp. (×1000 orginal magnification)

**Fig. 3:**
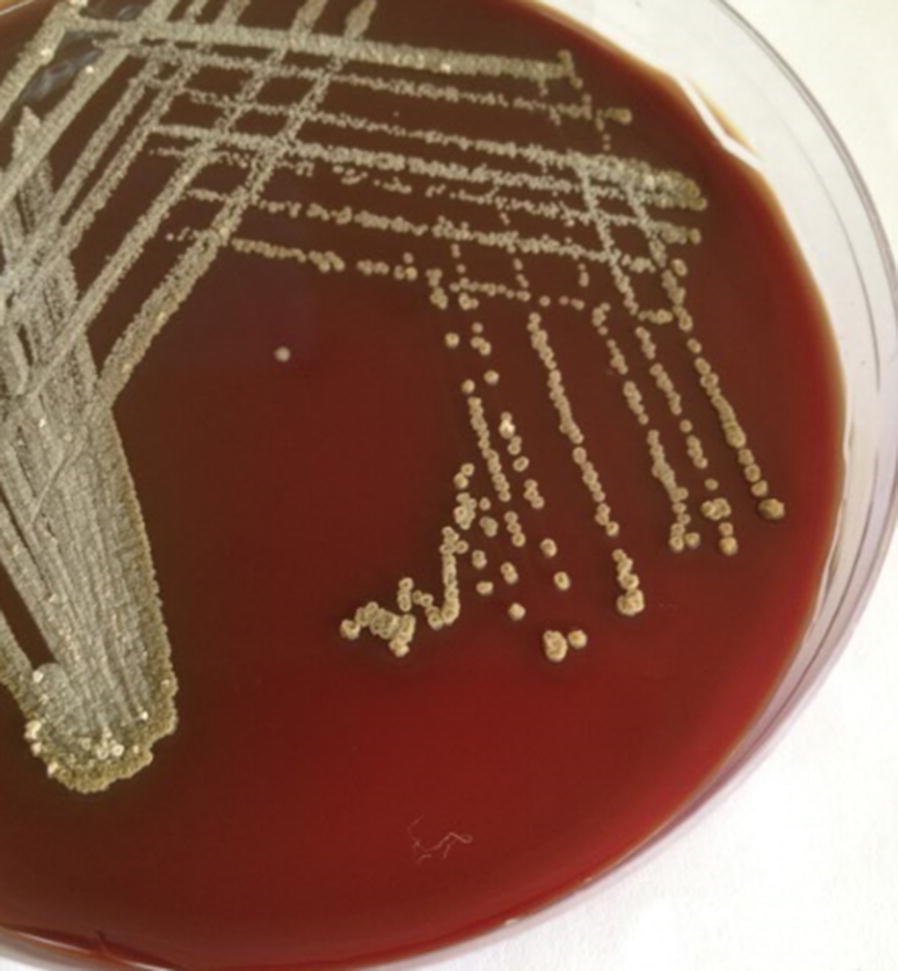
Colonial morphology of *Actinomyces israelii* on blood agar: whitish chalky adherent colonies with molar tooth appearance

**Fig. 4:**
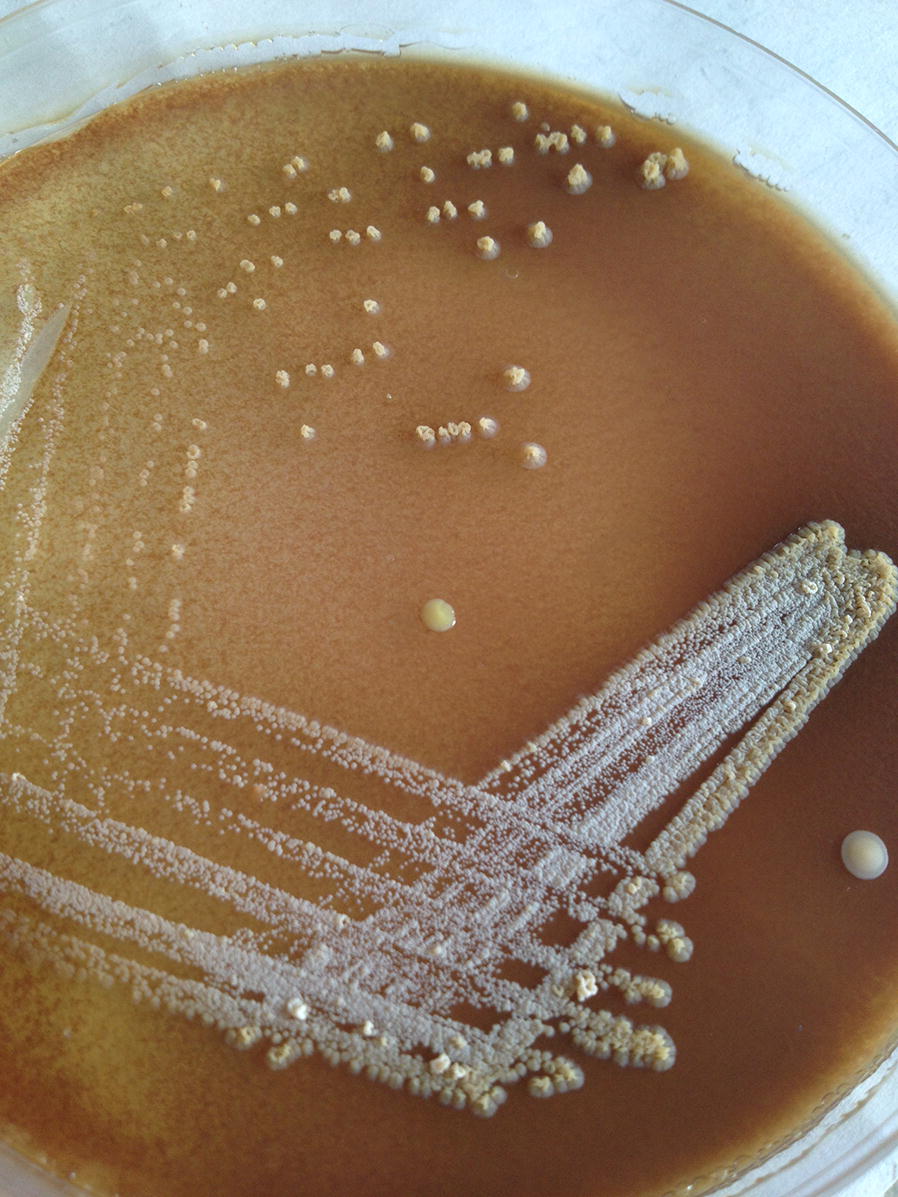
Colonial morphology of *Actinomyces israelii* on chocolate agar: whitish chalky adherent colonies with molar tooth appearance

The presumptive identification of etiologies was done with gram staining from the lesion which revealed branching filamentous gram-positive bacteria suggestive *Actinomyces* spp. (Fig. 2). As of further microbiological approaches, the cultured pus aspirate grew molar tooth shaped adherent colonies after 72 h of incubation at 36 °C on blood agar and chocolate agar in presence of ambient air, 5% Co_2_ (Figs. 3, 4). Since molecular analysis and sequencing was not accessible in our laboratory setting; further, identification of the isolate, *Actinomyces israeli*, was done with standard microbiological culture methods as recommended by American Society for Microbiology based upon phenotypic characteristics and biochemical interpretations [8]. In brief, colony morphology (chalky, matt, dry, crumbly, adherent in appearances; 0.5–2.0 mm in diameter with fine intertwining, branching filaments); in-house set of biochemical test: pigmentation (negative), catalase (negative), nitrate reduction (positive), hydrolysis of urea (negative) while esculin (positive), production of α-glucosidase and β-galactosidase (positive) while α-fucosidase and β-NAG (negative), fermentation of arabinose, maltose, raffinose, rhamnose, sucrose, xylose, trehalose (positive) while mannitol (negative). The antimicrobial susceptibility testing was done by modified Kirby-baur disc diffusion method on blood agar against commercially prepared antibiotic disks (Hi-Media Laboratories, Pvt, limited, India) in compliance with Clinical Laboratory Standards Institute (CLSI). The isolate was sensitive to penicillin G, amoxicillin, ceftriaxone, meropenem, doxycycline, linezolid, clindamycin, while ciprofloxacin and erythromycin were found resistant. Gene Xpert testing from the pus sample was negative for *Mycobacterium tuberculosis* with no associated resistant gene. No fungal elements grew from the pus aspirate; blood and urine sample were sterile.

Additionally, CT scan of abdomen and chest was done to rule-out possible disseminated actinomycosis; conversely, no abnormalities detected. In view of clinical manifestations and investigation reports, a diagnosis of primary cutaneous actinomycosis was made in HIV positive patient. After then, anti-tubercular therapy was discontinued and the patient was treated with intravenous benzylpenicillin (Penicillin G) for 6 weeks followed by oral phenoxymethylpenicillin (Penicillin V) for another 6 weeks.

## Treatment

The patient was treated with penicillin G–24 million U/d intravenous by continuous infusion for 6 weeks; and then shifted to oral penicillin V for another 6 weeks then to follow. The oral penicillin was continued up to 12 months.

## Outcomes and follow-up

**Fig. 5:**
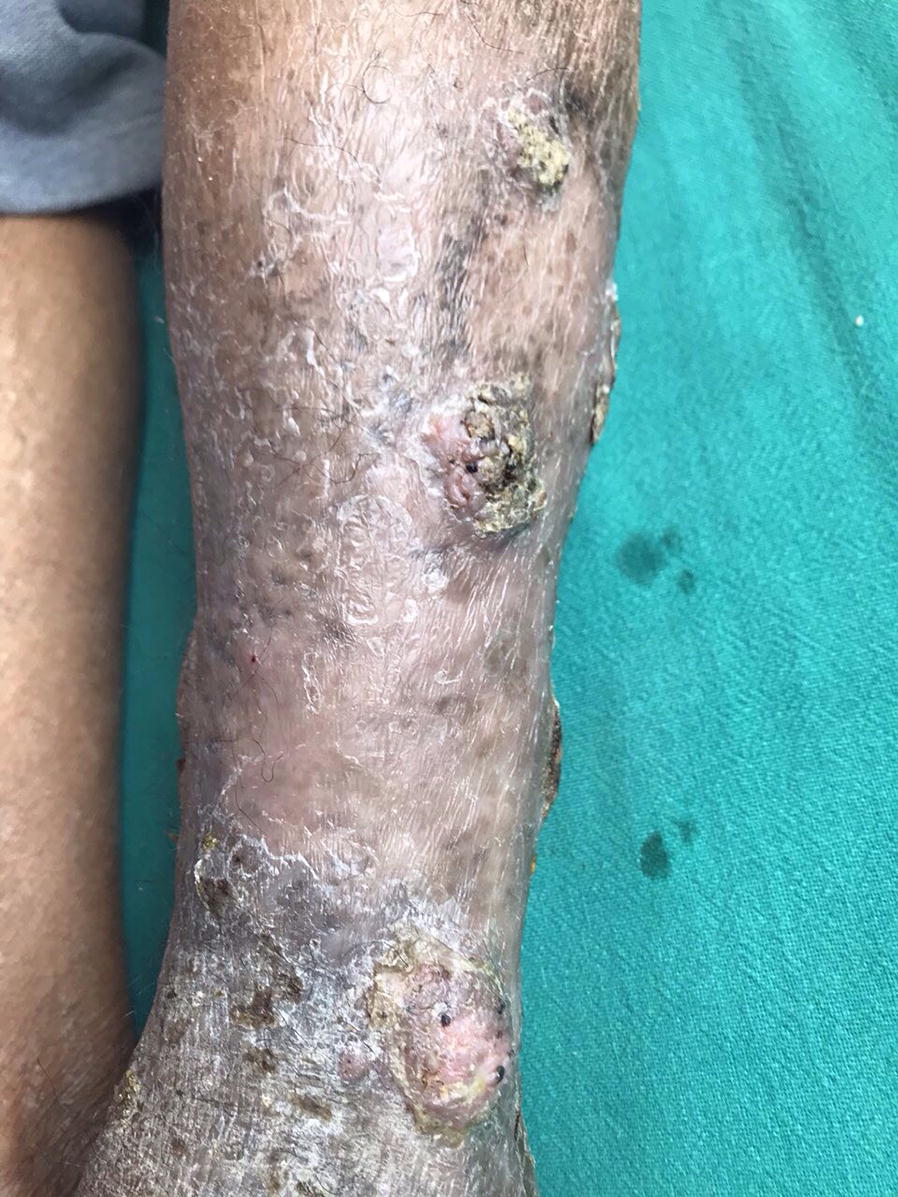
Flattening and regression of the indurated lesion after 3 months of antimicrobial therapy (Penicillin)

He has now completed 3 months of antimicrobial therapy; has undergone progressive changes—flattening and regression of the indurated lesion observed—no sign of relapse noted (Fig. 5). The oral penicillin V continued for a year to limit the possible late relapse. Now, the lesion healed completely without recur.

## Literature search

All the relevant information presented in table and literature review included in the manuscript was collected via searching Google, Pub Med/NCBI and other similar databases from 1986 to 2017. Key search terms were actinomycosis, HIV/AIDS, diagnosis consideration and clinical management (Table 1) [9–25].

**Table 1:** Summary of actinomycosis and its clinical management in patient living with HIV/AIDS patient, reported in English-language literature

S. no	References	CD4 count/µL	Surgical intervention	Antimicrobial therapy	Treatment duration	Outcomes
Cervicofacial actinomycosis						
1	Yeager et al. [9]	NA	Not done	Penicillin G (IV) + oral penicillin	Penicillin-G = 6 weeks; oral penicillin = 3 months	Cured
2	Watkins et al. [10]	NA	Edematous soft tissue and sequestrum were removed	Penicillin G (IV)	6 weeks	Cured
3	Manfredi et al. [11]	case 1(11); case 2(4)	Surgical debridement of tissue	Itraconazole + ceftazidime + netilmicin	Few weeks	Died
4	Kingdom et al. [12]	499	Not done	Oral penicillin	3 months	Cured
5	Manfredi et al. [13]	(case 1)9	Surgical debridement of tissue	Fluconazole. Ceftriaxone, netilmicin, itraconazole and ceftazidime	Few days	Died
		(case 2)2	Surgical debridement of tissue	Fluconazole, ceftriaxone, netilmicin, itraconazole, piperacillin and clindamycin	45 days	Died
6	Vazquez et al. [14]	480	Surgical incision of the tissue	Oral amoxycillin	3 weeks	Cured
7	Spadari et al. [15]	NA	Not done	Oral doxycycline (patient was penicillin allergic)	2 months	Died
8	Yuria et al. [16]	63	Not done	Intravenous ampicillin	2 weeks	Cured
9	Sudhakar et al. [17]	82	Not done	Ampicillin salbactam (IV) + amoxycillin–clavulanic acid	Ampicillin salbactam (IV) = 4 days; amoxycillin–clavulanic acid = 5 days	Cured
10	Klein et al. [18]	367	Removed maxillary bone sequestration	Penicillin G (IV) + amoxycillin	Penicillin G (IV) = 3 weeks; oral amoxicillin = 6 months	Cured
Thoracic actinomycosis						
11	Cendan et al. [19]	NA	Not done	Penicillin G	4 weeks	Died
Pulmonary actinomycosis						
12	Tabarsi et al. [20]	NA	Upper lobectomy	Oral penicillin; clindamycin, metronidazole, trimethoprim/sulfamethoxazole (TMP–SMX) and ceftriaxone were added to Highly Active Antiretroviral Therapy (HAART)	6 months	Cured
Gastro interstinal actinomycosis						
13	Spencer et al. [21]	NA	Not done	Antacids and ketoconazole	Not described	Cured
14	Litt et al. [22]	100	Not done	Oral penicillin	5 months	Cured
15	Arora et al. [23]	4	Not done	Intravenous penicillin + fluconazole	4 weeks	Died with other cause
16	Redelman et al. [24]	22	Not done	Doxycycline (IV) (the patient was allergic to penicillin)	4 months	Died with other cause
Primary cutaneous actinomycosis (lower extermities)						
17	Gomes et al. [25]	123	Wound debridement	Trimethoprim + sulfomethoxazole + ART (emtricitabina + tenofovir, ritonavir, and darunavir)	4 months	Cured

*ART* antiretroviral therapy, *IV* intra-venous, *HAART* highly active antiretroviral therapy, *TMP–SMX* trimethoprim/sulfamethoxazole

## Discussion

The emergence of HIV and the onset of AIDS epidemic have been associated with a myriad of opportunistic cutaneous infections; however, the cutaneous actinomycosis is out-of-the limelight from the differential diagnosis. The masquerading clinical presentations as cutaneous tuberculosis, fungal infections, malignancies and other systemic infections, difficult in vitro cultivation of the pathogen, and non-specific radiological picture are commonly associated outfits leading misdiagnosis [6]. In our case, the primordial diagnosis was made as cutaneous tuberculosis, based upon the clinical presentations and unresolved lesion with extended courses of antimicrobial therapy (cloxacillin). Further, unresolved lesion, even after the anti-tubercular therapy implies a differential diagnosis of rifampicin-resistant cutaneous tuberculosis, mycetoma/madura foot, and cutaneous nocardiosis.

Relating the endogenous habitat or colonization of the pathogen, *Actinomyces israelii*, the primary cutaneous actinomycosis of a lower extremity is extremely rare; associated either with post-traumatic exposure or direct implantation of the pathogen via animal, insects or human bites [26–28]. Linking the common ground of infection acquisition, probably the pathogen could have inoculated from the dog bites since no other clinical history suggesting sourced pathogen reported. No detectable extra-cutaneous lesions and radiological picture portentous to the dissemination were observed.

Moreover, difficulties in in vitro cultivation of the pathogen attribute further diagnostic challenges; since, longer incubation, up to 10 days, is obligatory prior to be reported as sterile [3, 29]. Earlier, the presumptive diagnosis could have made at least with gram staining in the local hospital; despite, relying only upon clinical manifestations; which inturn could prevent the erroneous diagnosis and treatment. Needless to say, but it is the ground reality of clinical practice, in developing countries like Nepal. The identification of the etiology, *Actinomyces israelii*, was done by standard microbiological culture methods as recommended by the American Society for Microbiology based upon: phenotypic characteristics of the isolate, its antimicrobial susceptibility pattern against antibiotics, extended incubation period and biochemical interpretations; sequencing of 16SrRNA, however, was not available in our setting [8, 29, 30]. Therefore, prior to starting the antimicrobial therapy, a high index of clinical suspicion together with close collaboration with microbiological interpretations is of utmost importance, for successful outcomes.

For successful management and treatment in cutaneous actinomycosis—limiting possible late relapse: early detection of the pathogen, recommended surgical debridement along with the appropriate selection of antimicrobial therapy, correct dosing, and treatment duration; are crucial [31, 32]. High-dose of penicillin over a prolonged period, 6 months to 1 year, is presumed as the drug of choice for all forms of actinomycosis [1, 6, 17, 27, 32]. As an alternative to penicillin, if the patient is hypersensitive to penicillin or if etiologies are resistant to penicillin, the antibiotics such as clindamycin, erythromycin, tetracycline, doxycycline, ceftriaxone, and chloramphenicol could be opted [32–34]. However, the clinicians must be cautious while opting the antibiotics: metronidazole, aminoglycosides, aztreonam, co-trimoxazole (TMP–SMX), penicillinase-resistant penicillins (e.g., methicillin, nafcillin, oxacillin, cloxacillin), and cephalexin, since these antibiotics possess nearly no activity against *Actinomyces* spp. [33.]. Unfolding to our case, no progressive changes on the lesion occurred; since as implicated antibiotic therapy i.e. cloxacillin was opted, initially. Although, on shifting to penicillin the patient undergone progressive changes; lesion healed subsequently and no relapse noted. Therefore, it is mandatory that the therapeutic regimen should be customized for each patient depending upon the susceptibility of the pathogen against the antibiotics.

The adverse reaction, nevertheless, owing to prolonged and high dose of penicillin therapy, are common including pseudomembranous colitis, interstitial nephritis, epigastric distress, urticarial, leukopenia, allergic reactions, eosinophilia, and super-infection [35]. No such side effects were found to be associated in our case, however. The patient reported a few episodes of nausea, vomiting, and diarrhea during 12 months of medication as minimal side effects.

## Conclusion

In clinical practice, the cutaneous actinomycosis often outfits with diagnostic challenges, owing to the multifaceted clinic-pathological features as of cutaneous infection with different etiology, and inherent difficulty in in vitro cultivating. Therefore, cutaneous actinomycosis requires a diagnosis consideration, especially in PLHA, where myriad of opportunistic cutaneous infections are common.

## Data Availability

All data generated or analyzed during this study are included in this published article.

## Abbreviations

ATT: anti-tubercular treatment
CLSI: Clinical and Laboratory Standard Institute
CT: computerized tomography
HBsAg: hepatitis B surface antigen
HCV: hepatitis C Virus
HIV: human immunodeficiency virus
PLHA: people living with HIV/AIDS
TMP–SMX: trimethoprim–sulfamethoxazole
